# Supplementary material for: Predicting visual function by interpreting a neuronal wiring diagram
Source: Nature. 2024 Oct 2;634(8032):113–23. doi: 10.1038/s41586-024-07953-5 (PMC11446822; doi:10.1038/s41586-024-07953-5)
Supplement: Supplementary file 5 — CRF and ERF predictions for individual Dm3 cells. The CRF is predicted for each target cell by mapping the top five monosynaptic pathways from hexel source types to the target Dm3 type. The ERF is predicted using disynaptic pathways that pass through the top ten intermediary types (ranking from Extended Data Fig. 7). As a set of disynaptic pathways passes from multiple hexel source types through each intermediary to the target, for brevity only one representative starting from a single hexel type is shown, the pathway in the set with maximal strength (red line, Extended Data Fig. 7). The centre of the Tm1–Dm3 CRF (Methods) is indicated by the cyan star, to aid comparison of locations across maps. Next to each map are the maximum and sum over all hexels, in units of 0.01%. In the last panel, all ellipse approximations are shown together for comparison, scaled up by ×3 relative to maps. The dashed ellipse approximates the Tm1–Dm3 CRF, and scale bars indicate one lattice constant and p and q axes. Each file name contains the ID of the target cell in v783. [file 41586_2024_7953_MOESM5_ESM.zip › DataS3/Dm3p/720575940626500670.pdf]

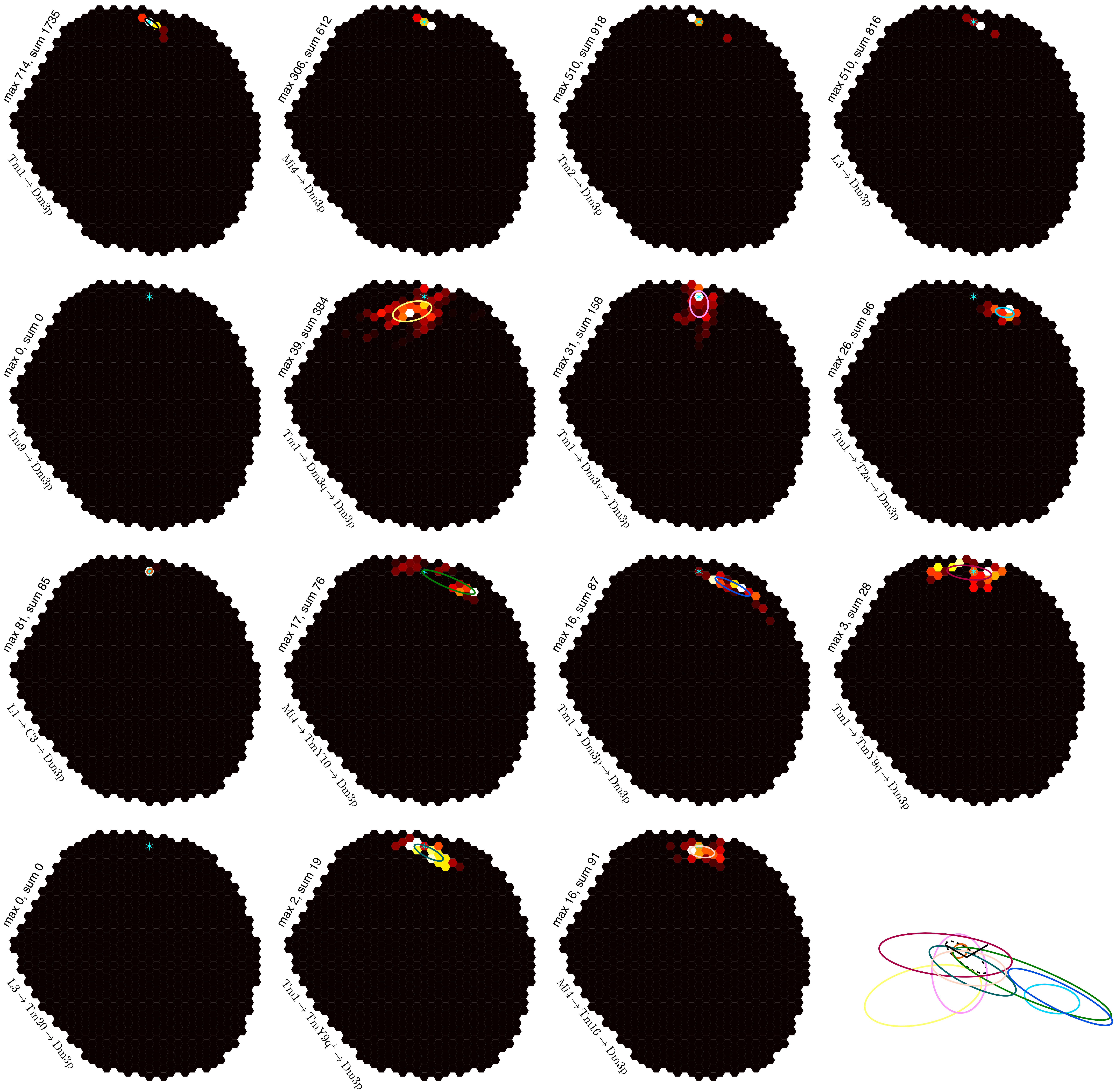
max 714, sum 1735
Tm1 → Dm3p
max 306, sum 612
Mi4 → Dm3p
max 510, sum 918
Tm2 → Dm3p
max 510, sum 816
L3 → Dm3p
max 0, sum 0
Tm9 → Dm3p
max 39, sum 384
Tm1 → Dm3q → Dm3p
max 31, sum 158
Tm1 → Dm3v → Dm3p
max 26, sum 96
Tm1 → T2a → Dm3p
max 81, sum 85
L1 → C3 → Dm3p
max 17, sum 76
Mi4 → TmY10 → Dm3p
max 16, sum 87
Tm1 → Dm3p → Dm3p
max 3, sum 28
Tm1 → TmY9q → Dm3p
max 0, sum 0
L3 → Tm20 → Dm3p
max 2, sum 19
Tm1 → TmY9q⊥ → Dm3p
max 16, sum 91
Mi4 → Tm16 → Dm3p
